# The Effect of 3-Week Betaine Supplementation on Blood Biomarkers of Cardiometabolic Health in Young Physically Active Males

**DOI:** 10.3390/metabo12080731

**Published:** 2022-08-07

**Authors:** Emilia Zawieja, Krzysztof Durkalec-Michalski, Agata Muzsik-Kazimierska, Agata Chmurzynska

**Affiliations:** 1 Department of Human Nutrition and Dietetics, Poznan University of Life Sciences, 60-624 Poznan, Poland; 2 Department of Sports Dietetics, Poznan University of Physical Education, 61-871 Poznan, Poland; 3 Department of Physiology and Biochemistry, Faculty of Physical Education and Sport, Charles University, 162 52 Prague, Czech Republic

**Keywords:** betaine, blood lipids, MTHFR, homocysteine, cardiometabolic health, physical activity

## Abstract

Betaine (BET) supplementation decreases homocysteine concentration in plasma, but it may also have an adverse effect on health by increasing blood lipid concentrations, at least in overweight and obese individuals. The aim of this study was to evaluate the effect of BET supplementation on the lipid profile and concentrations of homocysteine, inflammatory cytokines, and liver enzymes in physically active, healthy males. This was a randomized, placebo (PL)-controlled, double-blinded, crossover trial. BET (2.5 or 5.0 g/d) was administered for 21 days. Before and after supplementation with BET or PL, anthropometric measurements and blood were collected in a fasted state. Our results show that BET supplementation significantly decreased homocysteine concentration (from 17.1 ± 4.0 μmol/L before BET to 15.6 ± 3.5 μmol/L after BET, *p* = 0.009, η^2^ = 0.164). However, the intervention had no effect on total cholesterol, low-density lipoprotein cholesterol, high-density lipoprotein cholesterol, triacylglycerol, interleukins 1β and 6, and tumour necrosis factor α concentrations, or alanine and aspartate activities. In addition, there were no interactions between the *MTHFR* genotype and BET dose. In conclusion, BET supplementation may be beneficial for homocysteine concentration in healthy, physically active males, with no detrimental effect on lipid profile.

## 1. Introduction

Betaine (BET) is a natural substance abundant both in plant and animal tissues. BET is present in food such as grains or vegetables, synthesized endogenously from choline, or ingested as a food supplement (due to its postulated beneficial properties). BET induces a reduction of homocysteine concentration in the blood [1], which may be a desirable effect, as homocysteine is a non-proteogenic amino acid, which has been associated with increased risk of cardiovascular diseases (CVD) [2,3], cognitive impairment and dementia [4], neurodegenerative diseases [5], bone disease, and neural tube defects in foetuses [6,7]. BET can also affect liver functioning by a variety of molecular mechanisms, including the inhibition of inflammatory response, improvement of insulin resistance, reduction of endoplasmic reticulum stress, alleviation of liver oxidative stress, increase in autophagy, remodelling of intestinal flora, and regulation of epigenetic modification [8]. 

Apart from its many health benefits, there are also data on the deleterious effect of BET on blood lipids. Olthof et al. [9] reported that BET leads to an increase in total cholesterol (TC) and low-density lipoprotein cholesterol (LDL-C) concentrations in a dose-dependent way, which could increase the risk for CVD. Our previous meta-analysis showed that BET had no effect on LDL-C, high-density lipoprotein cholesterol (HDL-C), or triacylglycerol (TAG) concentrations, but moderately increased TC [10]. However, this effect was especially seen in people with obesity. The mechanism responsible for the influence of BET on lipid profile is not fully understood, but BET may, for example, increase lipid export from the liver to the blood, thanks to its sparing effect on choline [11]. Since BET is synthesized endogenously from choline, it is possible that BET supplementation leaves more choline available for phosphatidylcholine biosynthesis in the liver. Phosphatidylcholine is necessary for very-low-density lipoprotein (VLDL-C) secretion, and VLDL-C is the precursor for LDL-C. Hence, BET may indirectly influence lipoprotein concentration in the blood. Another recent meta-analysis showed that BET increased both TC and LDL-C [12]. It is, however, unclear whether this effect would be observed in young, healthy, and athletic males with normal body mass index.

Another of the suggested beneficial effects of BET relates to its anti-inflammatory action. Inflammation is a natural immune reaction necessary to defend the human organism against pathogens and injuries. However, prolonged or excessive inflammatory reaction may lead to various diseases, including CVD. Macrophages are the first line of defence, identifying infections and then releasing cytokines and chemokines to recruit additional immune cells [13]. Notably, BET has been shown to be effective against many inflammatory diseases (e.g., diabetes and non-alcoholic fatty liver disease) [14]. BET inhibits nuclear factor-κB activity and NLRP3 inflammasome activation, and may hence influence the concentration of cytokines, such as interleukin 1β (IL-1β), interleukin 6 (IL-6), and tumour necrosis factor α (TNFα) [15].

Another well-known role for BET in the body is its involvement in one-carbon metabolism, including homocysteine methylation to methionine. Under normal conditions, methyl groups necessary for this reaction can derive from either 5,10-methylenetetrahydrofolate or BET. However, people deficient in folate or with impaired folate metabolism cannot effectively use both methyl sources and may need more BET for proper functioning [16]. Impaired folate metabolism may result from a single nucleotide polymorphism (SNP) in the methylenetetrahydrofolate reductase (*MTHFR*) gene. MTHFR is an enzyme responsible for the transformation of 5-methyltetrahydrofolate to 5,10-methylenetetrahydrofolate, which is then used in the homocysteine-methionine cycle. *MTHFR* C677T SNP (rs1801133) may influence this reaction because the T-allele carriers synthesize thermolabile enzymes with lower activity [17]. Thus, T-allele carriers would, hypothetically, need more BET than CC homozygotes. However, to the best of our knowledge, there are no studies investigating the differences in response to BET supplementation related to *MTHFR* genotype.

The aim of our study was to evaluate the effect of 3-week BET supplementation (2.5 and 5.0 g/d) on the concentrations of homocysteine, blood lipids, glucose, liver enzymes, and inflammatory cytokines, in a group of young, healthy, and physically active males. We also investigated the interaction between BET treatment, its dosage (2.5 vs. 5.0 g/d), and the *MTFHR* genotype. We hypothesized that T-allele carriers will respond differently to CC homozygotes, and that 5.0 g/d BET will induce greater changes in outcome measures than 2.5 g/d BET.

## 2. Materials and Methods

### 2.1. Study Design

This study was designed in a randomized crossover placebo-controlled double-blinded fashion. The participants ingested both BET and placebo (PL) for 3 weeks in a random order. Supplementation periods were separated by a 3-week washout. Before and after each supplementation period (i.e., before BET (BET_pre_), after BET (BET_post_), before PL (PL_pre_), and after PL (PL_post_)), blood samples were drawn and body composition was measured. Participants were instructed not to introduce any changes in their lifestyle and diet throughout the entire study period. Participants were divided into two groups, one receiving 2.5 g/d, and the other receiving 5.0 g/d. Participants were also divided post hoc into groups according to *MTHFR* genotype (T-allele carriers and CC homozygotes). The study protocol was registered at https://clinicaltrials.gov/ct2/show/NCT03702205 (accessed on 30 June 2022). All samples were collected from January 2019 to December 2021. The study was conducted at the Department of Human Nutrition and Dietetics, Poznan University of Life Sciences, Poland. The randomization was performed using https://studyrandomizer.com (accessed on 20 August 2021) by an impartial person, and group prescription was unblinded only after the end of the study. The study was approved by the local ethical committee (Bioethics Committee at Poznan University of Medical Sciences, Poznan, Poland. Decision no. 1092/17 of 9 November 2017), and written informed consent was obtained from all participants before the study began. All procedures were conducted in accordance with the ethical standards of the 1964 Helsinki Declaration.

### 2.2. Participants

**Figure 1:**
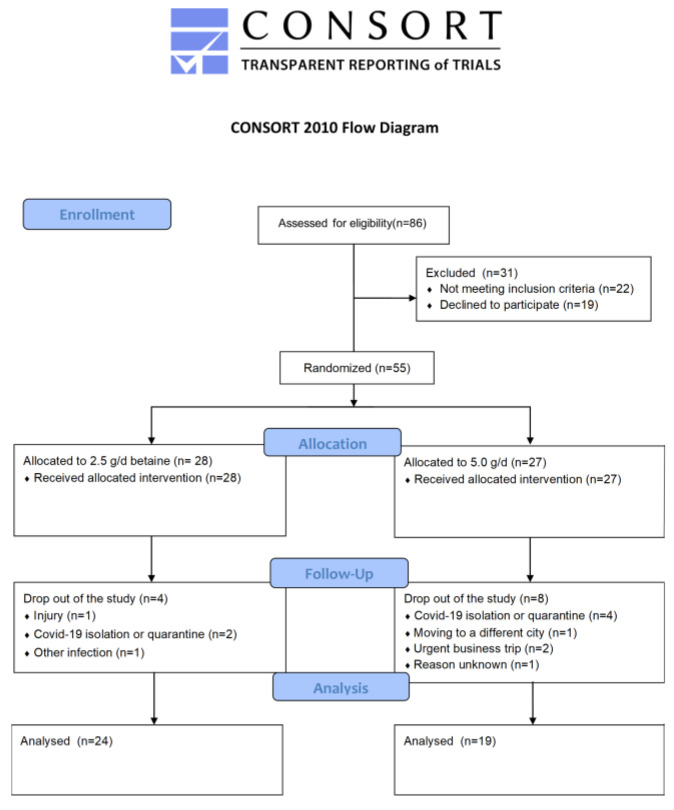
Consort flow diagram.

Inclusion criteria were as follows: males, aged between 18 and 45 years, currently training CrossFit for at least 1 year, in good health condition. Exclusion criteria included injury or chronic disease, and supplementation with vitamin B, BET, or choline for at least 3 months before participating in the study. The sample size was determined a priori using G*Power for repeated measures within/between ANOVA. A total of 36 participants was necessary to achieve 96% chance of correctly rejecting the null hypothesis with medium effect size (η^2^ = 0.06), α = 0.05 and power set to 95%. Out of 55 initially recruited participants, 43 finished the whole study protocol. Reasons for dropping out were as follows: injury (*n* = 1), moving to a different city (*n* = 1), urgent business trip (*n* = 2), COVID-19 quarantine or isolation (*n* = 6), other infections (*n* = 1), reason unknown (*n* = 1) (Figure 1). Mean age was 34.2 ± 6.1 years, mean height 178.7 ± 6.0 cm, mean BM 82.0 ± 9.5 kg, and mean body fat 17.6 ± 6.7%.

### 2.3. Supplementation

BET was supplemented in one of two daily doses: 2.5 or 5.0 g. BET was administered in white cellulose capsules containing 500 mg BET. PL was cellulose in similarly looking white capsules. Both BET and PL were packed in white containers with the same aspect. Supplements were provided by Medicaline Konrad Malitka, Karczew, Poland. Participants with 2.5 g/d BET ingested 5 capsules per day (3 in the morning and 2 in the evening), and those with 5.0 g/d BET ingested 10 capsules per day (4 in the morning, 3 in the afternoon, and 3 in the evening). The capsules were ingested with at least 250 mL of water. No side effects were reported. The adherence to supplementation was monitored with patient surveys. Moreover, at the end of each supplementation period, the participants were asked to bring back the bottle/s with the remaining capsules. The initial number of capsules was 105 per bottle. Participants in a 2.5 g/d group received one bottle (5 capsules per day, 21 days) and participants in a 5.0 g/d group received two bottles (10 capsules per day). If there were any capsules left at the end of the supplementation period, it would indicate not adhering to the supplementation regimen.

### 2.4. Body Composition

Body composition was measured upon fasting in the morning, based on air displacement plethysmography, and using the Bod Pod^®^ (Cosmed, Rome, Italy). Once body density was determined, the FM (FM) and fat-free mass (FFM) were calculated using the Siri equation. Thoracic lung volume was estimated using the Bod Pod^®^ software. During the measurement, participants wore only a swimsuit and an acrylic swim cap. Repeatability of Bod Pod measurements was previously determined as high [18].

### 2.5. Biochemical Analysis

Blood samples (BET_pre_, BET_post_, PL_pre_, and PL_post_) were collected fasted in the morning by a qualified person. After collection, serum was obtained by centrifugation of venous blood clots at 1000 rpm for 10 min. The biological material was stored frozen at −80 °C until analysis. The levels of TC, LDL-C, HDL-C, TG, glucose, ALAT, and ASPAT were determined using an automated analyser system (Konelab20i biochemical analyser, ThermoElectron Corporation, Vantaa, Finland). 

### 2.6. Concentrations of Cytokines and Homocysteine

IL-1β, Il-6, and TNFα concentrations were measured in plasma by ELISA using commercially available kits. The following kits were used: TNF alpha Human ELISA Kit, Ultrasensitive (Catalog # KHC3014) (Invitrogen, Thermofisher Scientific, Vienna, Austria), IL-1 beta Human ELISA Kit (Catalog # KHC0012) (Invitrogen, Thermofisher Scientific, Vienna, Austria), IL-6 Human ELISA Kit (Catalog # KHC0061) (Invitrogen, Thermofisher Scientific, Vienna, Austria). Total reduced homocysteine concentration was measured using a fluorometric method with a commercially available kit (Catalog # MAK354-1KT) (Merck Life Science Sp. z.o.o., St. Louis, MO, USA), whereby the absorbance and fluorescence were measured with a microplate reader (Infinite Pro 200, Tecan, Austria).

### 2.7. MTHFR Genotyping

Blood samples for *MTFHR* (rs180113) genotyping were collected on the first study meeting in the morning. DNA was isolated from blood lymphocytes using a standard kit (NucleoSpin^®^ Blood, Mercherey-Nagel, Germany). Genotyping was performed using TaqMan probes (single-tube assays; Thermo Scientific, Waltham, MA, USA, assay ID C_1202883_20) on a LightCycler 480 instrument (Roche Diagnostics, Switzerland).

### 2.8. Statistical Analysis

The normality of the data was assessed by the Shapiro–Wilk test. Baseline differences between groups were measured using a Student’s *t*-test. Participants were divided into two groups regarding median fat mass (FM). Correlations between outcomes were measured using the Pearson’s method. A series of within/between-subjects repeated measures analysis of variance (ANOVA) was used to compare concentrations of blood lipids, glucose, liver enzymes, and inflammatory cytokines. The within-factors were treatment (BET and PL) and time (pre- and post-supplementation). The between-factors were *MTHFR* genotypes (T-allele and CC homozygotes) and BET dose (2.5 and 5.0 g/d). For all measured variables, the estimated sphericity was tested with Mauchly’s W, and the Greenhouse–Geisser correction was used when necessary. All analyses were completed using SPSS Version 22 (IBM, Armonk, NY, USA), and an alpha level of <0.05 was set a priori.

## 3. Results

### 3.1. Baseline Differences

There were no baseline differences between BET dose groups (Table 1).

There were no differences in any outcome at baseline between *MTHFR* gene CC homozygotes and T-allele carriers (Table 2). Significant differences were found for fat mass (FM). Participants with FM > 16.2% had higher TAG, TC, and LDL-C concentrations than participants with FM < 16.2% (Table 2).

### 3.2. Correlations between Outcomes

TAG was significantly positively correlated with body mass (BM), FM (% and kg), TC, and LDL-C, but negatively correlated with FFM (%) (Table 3). There was a positive association between TC and FM (%), glucose, LDL-C, and TNFα, and a negative relationship between TC and FFM (% and kg). Besides TAG and TC, LDL-C was positively correlated with FM (% and kg) and negatively correlated with FFM (%). HDL-C was negatively correlated with TAG, BM, and IL-1β. Homocysteine was significantly positively correlated with FM (%) and negatively correlated with FFM (%).

### 3.3. The Effect of BET Supplementation on the Concentrations Blood Lipids, Glucose, and Homocysteine

**Figure 2:**
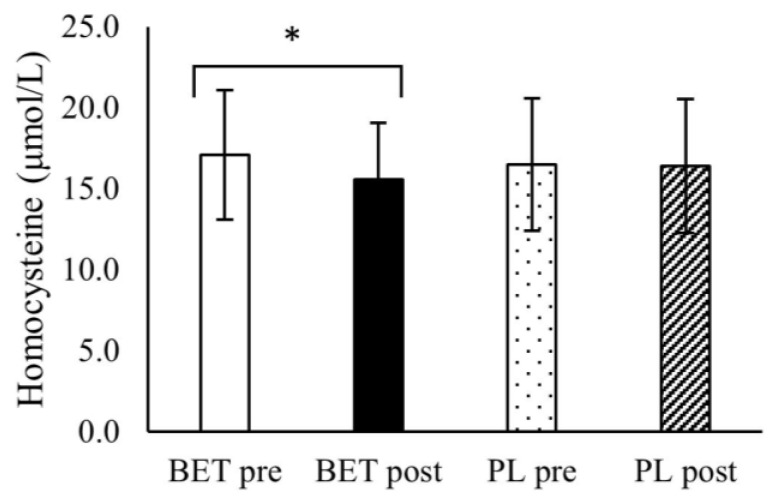
The effect of betaine supplementation on homocysteine concentration. BET_post_, after betaine supplementation; BET_pre_, before betaine supplementation; PL_post_, after placebo supplementation; PL_pre_, before placebo supplementation, * BET_pre_ vs. BET_post_ p value= 0.020.

There was a significant interaction between time and treatment for homocysteine concentration (*p* = 0.009, η^2^ = 0.164). Homocysteine concentration was significantly lower after BET vs. before BET (Figure 2).

**Figure 3:**
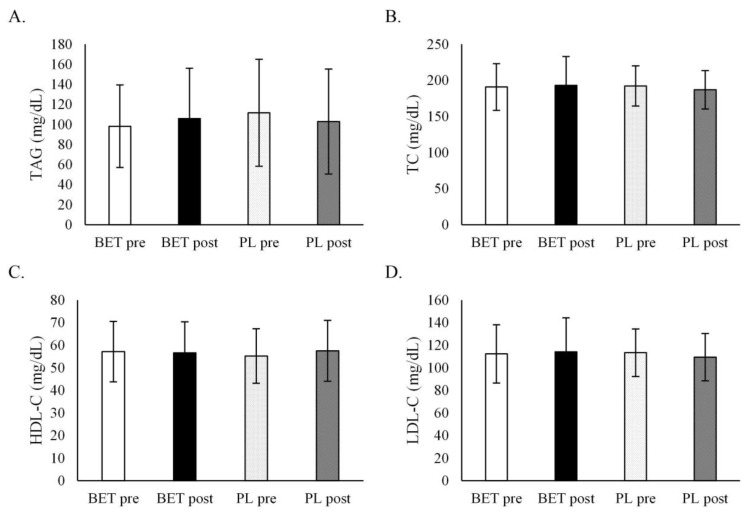
The effect of betaine supplementation on blood lipids concentrations. (**A**). The effect of betaine supplementation on triacylglycerol concentration. (**B**). The effect of betaine supplementation on total cholesterol concentration. (**C**). The effect of betaine supplementation on HDL cholesterol concentration. (**D**). The effect of betaine supplementation on LDL cholesterol concentration. BET_post_, after betaine supplementation; BET_pre_, before betaine supplementation; HDL-C, high-density lipoprotein cholesterol; LDL-C, low-density lipoprotein cholesterol; PL_post_, after placebo supplementation; PL_pre_, before placebo supplementation; TAG, triacylglycerol; TC, total cholesterol.

BET had no influence on blood lipids, i.e., TC, TAG, LDL-C, HDL-C, and glucose concentrations (Table 4, Figure 3). There was no interaction with *MTHFR* genotype or BET dose.

**Figure 4:**
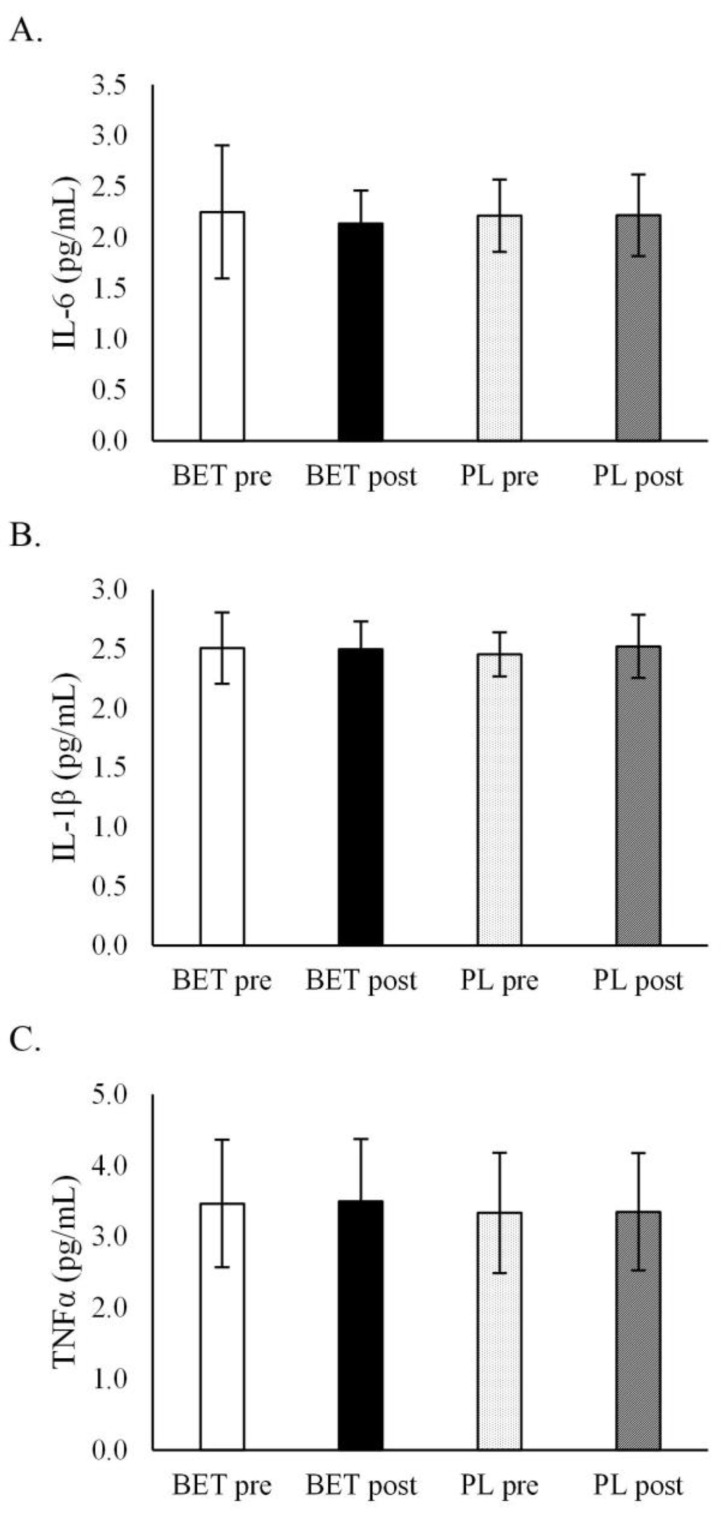
The effect of betaine supplementation on cytokines concentrations. (**A**). The effect of betaine supplementation on interleukin 6 concentration. (**B**). The effect of betaine supplementation on interleukin 1β concentration. (**C**). The effect of betaine supplementation on tumour necrosis factor α concentration. BET_post_, after betaine supplementation; BET_pre_, before betaine supplementation; IL, interleukin; PL_post_, after placebo supplementation; PL_pre_, before placebo supplementation; TNF-α, tumour necrosis factor α.

BET had no influence on inflammatory cytokine concentrations (Table 5, Figure 4). There was no interaction with *MTHFR* genotype or BET dose.

**Figure 5:**
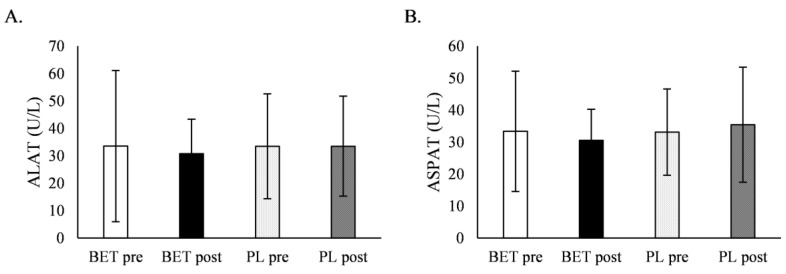
The effect of betaine supplementation on liver enzymes. (**A**). The effect of betaine supplementation on alanine transferase. (**B**). The effect of betaine supplementation on aspartate transferase. ALAT, alanine transferase; ASPAT, aspartate transferase; BET_post_, after betaine supplementation; BET_pre_, before betaine supplementation; PL_post_, after placebo supplementation; PL_pre_, before placebo supplementation.

There was no effect of BET on liver enzymes (Table 6, Figure 5), and no interaction with *MTHFR* genotype or BET dose. 

## 4. Discussion

Several previous studies have investigated the influence of BET supplementation on the concentrations of homocysteine, blood lipids, liver enzymes, and pro-inflammatory cytokines. The present study is, however, the first to evaluate the effect of 21-day BET supplementation in physically active, healthy males. We observed decreased homocysteine concentration after BET supplementation, and no effect of the intervention on the concentrations of TC, TAG, HDL-C, LDL-C, and glucose, as well as ALAT and ASPAT. There was also no influence of BET on IL-1β, IL-6, or TNFα. Moreover, the effect of BET supplementation was not dependent on *MTHFR* genotype, and no difference was observed between 2.5 and 5.0 g/d BET doses. However, our results might indicate a slight benefit from BET supplementation in case of cardiometabolic health connected with lower homocysteine concentration, with no other side effects in active and healthy males.

One of the best described benefits of BET is a decrease in homocysteine concentration, which is associated with lower risk of CVD [19]. Our results confirm this statement. In our study, homocysteine concentration was not different at baseline in T-allele carriers and CC homozygotes, which is opposite to previous studies [20]. Interestingly, our participants had mildly elevated homocysteine concentrations (above 15.0 μmol/L). Blood homocysteine concentration depends mainly on gene polymorphisms (e.g., *MTHFR*) and/or availability of vitamin B12 and folate [21]. Homocysteine can be remethylated to methionine, using a methyl group from N-5-methyltetrahydrofolate or from BET. Previous studies showed that BET supplementation lowers homocysteine concentration in blood [18]. We also showed that 2.5 g/d of BET was enough to cause this effect, with no further benefit from 5.0 g/d of BET.

A previous study by Olthof et al. [9] suggested that the benefit from BET supplementation in case of cardiometabolic health may be negated by a concomitant increase in TC and LDL-C levels. However, our study showed that BET is safe for the lipid profile in young and healthy males. McGregor et al. [22] found that BET combined with folate and vitamin B6 raised TC (by 7.2%), HDL-C (by 4.7%), LDL-C (by 10.3%), and TAG (by 10.4%) compared to folate and B6 alone, after a three-month treatment in patients with renal failure. Schwab et al. [23] showed that people with obesity had significantly increased TC (by 12.2%) and LDL-C (by 23.3%) concentrations after 16-week BET supplementation combined with hypo-energetic diet compared to the control group (hypo-energetic diet alone). Olthof et al. [9] assessed the effect of BET on blood lipids in healthy participants. They showed an increase in TC (by 8%) and LDL-C (by 11%) after six weeks of BET treatment. Tiihonen et al. [24] observed an increase in HDL-C concentration after 12-week BET supplementation, but there were no differences in TC and LDL-C in Asian males with mild fatty liver disease. Grizales et al. [25] observed increased TC with BET (10-day supplementation) compared to placebo, with no change in TAG, LDL-C, and HDL-C in people with obesity and prediabetes. It is important to notice that most of the studies investigating the effect of BET on blood lipids recruited participants suffering from obesity and other metabolic diseases. Two meta-analyses also confirmed BET’s deleterious effect on blood lipids [10,12]. However, as shown in our study, this effect was not observed in healthy, physically active males ingesting 2.5 and 5.0 g/d BET. The effect of BET on blood lipids that was observed in some patients can be attributed to the increased synthesis of very low-density lipoproteins in the liver, which are the precursors for LDL-C [26]. This effect could be more pronounced in people with accumulated lipids in the liver, or decreased phosphatidylethanolamine N-methyltransferase activity (e.g., due to alcohol abuse).

*MTHFR* T-allele carriers were associated with higher TC and LDL-C by a recent meta-analysis, but we did not register any significant differences at baseline in the concentrations of blood lipids between T-allele carriers and CC homozygotes [27]. There was also no interaction between *MTHFR* genotype and BET treatment. Blood lipids were correlated mostly with body mass and composition. Higher FM was associated with higher TAG, TC, and LDL-C. Participants with FM > 16.2% had higher TAG, TC, and LDL-C concentrations than leaner participants. It is well known that FM is related to lipid profile disturbances, even in subjects with normal weight, and this was in agreement with our results [28]. 

Our study showed no influence of BET on liver enzymes. In humans, serum ALAT and ASPAT activities are commonly measured clinically, as biomarkers of liver health. A recent review showed that BET improves liver function by a variety of molecular mechanisms, i.e., inhibition of inflammatory response, improvement of insulin resistance, reduction of endoplasmic reticulum stress, alleviation of liver oxidative stress, increase of autophagy, remodelling of intestinal microbiota, and regulation of epigenetic modification [8]. Nevertheless, the meta-analysis showed that BET has no impact on ALAT and ASPAT, which is in agreement with our results. Generally, liver and kidneys are the organs with more abundant BET (about 4097 μmol/L and 3785 μmol/L, respectively, as shown in rats) [29]. In this respect, BET’s major role in the liver is as a methyl-group donor. Liver biopsies showed decreased steatosis grade in patients with non-alcoholic liver disease [30]. The reason for lack of effect of BET on ALAT and ASPAT may be because they do not reflect the function of the liver, but rather hepatocellular or muscle injury [31].

BET may also influence CVD risk by lowering the inflammation indices [16]. However, we did not observe any change in inflammatory cytokines after 3-week BET supplementation, which is in agreement with the study by Abdelmalek et al. [30] in patients with non-alcoholic fatty liver disease. However, in contrast with our investigation, Nobari et al. [32] showed reduced levels of TNFα, IL1β, and IL-6 after 14-week BET supplementation in young soccer players. The majority of studies on BET’s influence on inflammatory indices were performed on animals, and the effect in humans is still to be determined.

Our study is not without limitations. The greater one is a lack of blood BET, trimethylamine-N-oxide, choline, vitamin B12, and folate concentrations measurements. Vitamin B12 and folate play an important role in homocysteine to methionine metabolism. Baseline BET concentration could have also influenced the effect of the intervention. Additionally, the BET doses were not adjusted to body mass.

## 5. Conclusions

In conclusion, the results of the present study provide evidence that BET supplementation may have a small positive effect on cardiometabolic health, by decreasing homocysteine concentration. However, this statement is speculative and further studies are necessary to establish a causative effect of BET supplementation on CVD risk in different populations. Moreover, it appears that BET is safe for the lipid profile in a group of healthy, physically active males. Contrary to our hypothesis, BET action was not dependent on *MTHFR* genotype or supplemented BET dose. It appears that 2.5 g/d was enough to induce a decrease in homocysteine concentration.

## Figures and Tables

**Table 1:** Baseline characteristics of experimental groups.

	BET Dose		*t*-Test
	2.5 g/d (*n* = 24) Mean ± SD	5.0 g/d (*n* = 19) Mean ± SD	*p*-Value
Age (years)	34.1 ± 6.0	34.2 ± 6.4	0.964
Height (cm)	179 ± 7.0	178 ± 4.7	0.587
Body mass (kg)	81.9 ± 10.0	82.7 ± 9.0	0.765
TAG (mg/dL)	109.5 ± 52.3	112.5 ± 49.5	0.851
TC (mg/dL)	197.3 ± 30.7	191.6 ± 29.0	0.546
LDL-C (mg/dL)	117.8 ± 21.9	111.2 ± 25.2	0.369
HDL-C (mg/dL)	54.9 ± 11.8	55.7 ± 14.3	0.845
Glucose (mg/dL)	87.9 ± 8.0	89.2 ± 8.8	0.603
ALAT (U/L)	37.1 ± 37.7	32.4 ± 17.2	0.614
ASPAT (U/L)	33.0 ± 20.2	34.2 ± 16.3	0.844
IL-6 (pg/mL)	2.16 ± 0.28	2.31 ± 0.40	0.159
IL-1β (pg/mL)	2.58 ± 0.34	2.48 ± 0.23	0.289
TNF-α (pg/mL)	3.35 ± 0.82	3.19 ± 0.84	0.531
Homocysteine (μmol/L)	16.5 ± 4.2	16.2 ± 3.8	0.762

ALAT, alanine transferase; ASPAT, aspartate transferase; FM%, percentage of fat mass; HDL-C, high-density lipoprotein cholesterol; IL, interleukin; LDL-C, low-density lipoprotein cholesterol; TAG, triacylglycerol; TC, total cholesterol; TNF-α, tumour necrosis factor α.

**Table 2:** Baseline concentrations for blood lipids, glucose, cytokines, homocysteine, and liver enzymes per *MTHFR* genotype and fat mass.

	*MTHFR*		*t*-Test	%FM		*t*-Test
	CC Homozygotes (*n* = 23) Mean ± SD	T-Allele Carriers (*n* = 20) Mean ± SD	*p*-Value	<16.2 (*n* = 21) Mean ± SD	>16.2 (*n* = 22) Mean ± SD	*p*-Value
TAG (mg/dL)	102.8 ± 45.8	120.3 ± 55.5	0.135	96.9 ± 36.0	138.4 ± 64.3	0.005
TC (mg/dL)	192.0 ± 30.6	184.8 ± 53.2	0.294	189.4 ± 31.5	205.8 ± 23.0	0.046
LDL-C (mg/dL)	113.6 ± 21.7	116.7 ± 25.6	0.338	110.2 ± 23.8	124.7 ± 19.7	0.028
HDL-C (mg/dL)	54.5 ± 11.5	56.2 ± 14.5	0.332	57.4 ± 13.2	51.0 ± 11.1	0.064
Glucose (mg/dL)	88.8 ± 8.5	83.8 ± 14.4	0.084	87.5 ± 9.2	90.3 ± 5.8	0.157
ALAT (U/L)	37.4 ± 39.9	32.2 ± 12.4	0.289	37.0 ± 35.9	30.9 ± 11.5	0.270
ASPAT (U/L)	33.7 ± 23.7	33.4 ± 10.0	0.475	34.8 ± 21.7	30.9 ± 8.3	0.259
IL-6 (pg/mL)	2.21 ± 0.43	2.25 ± 0.21	0.329	2.20 ± 0.28	2.28 ± 0.45	0.238
IL-1β (pg/mL)	2.55 ± 0.38	2.51 ± 0.17	0.329	2.53 ± 0.34	2.55 ± 0.18	0.401
TNF-α (pg/mL)	3.30 ± 0.82	3.26 ± 0.84	0.446	3.20 ± 0.83	3.46 ± 0.80	0.165
Homocysteine (μmol/L)	15.6 ± 4.7	17.2 ± 2.8	0.094	15.5 ± 3.2	17.3 ± 4.7	0.064

ALAT, alanine transferase; ASPAT, aspartate transferase; FM%, percentage of fat mass; HDL-C, high-density lipoprotein cholesterol; IL, interleukin; LDL-C, low-density lipoprotein cholesterol; MTHFR, methylene tetrahydrofolate reductase; TAG, triacylglycerol; TC, total cholesterol; TNF-α, tumour necrosis factor α.

**Table 3:** Correlations between baseline characteristics.

	Pearson R^2^	*p*-Value
**Correlations of TAG with**		
BM	+0.36	0.019
FM (%)	+0.38	0.014
FFM (%)	−0.38	0.014
FM (kg)	+0.42	0.006
TC	+0.32	0.038
LDL-C	+0.31	0.043
**Correlations of TC with**		
FM (%)	+0.37	0.015
FFM (%)	−0.37	0.015
FFM (kg)	−0.45	0.003
Glucose	+0.57	<0.001
LDL-C	+0.68	<0.001
TNF-α	+0.37	0.016
**Correlations of LDL-C with**		
FM (%)	+0.34	0.048
FFM (%)	−0.34	0.048
FM (kg)	+0.42	0.006
**Correlations of HDL-C with**		
BM	−0.32	0.047
TAG	−0.35	0.021
IL-1β	−0.32	0.045
**Correlations of homocysteine with**		
FM (%)	+0.328	0.032
FFM (%)	−0.328	0.032

BM, body mass; FFM, fat-free mass; FM, fat mass; HDL-C, high-density lipoprotein cholesterol; IL, interleukin; LDL-C, low-density lipoprotein cholesterol; TAG, triacylglycerol; TC, total cholesterol; TNF-α, tumour necrosis factor α.

**Table 4:** The effect of betaine supplementation on lipid profile, as well as homocysteine and glucose concentrations in regard to *MTHFR* genotype and dose of betaine.

		*MTHFR*		Dose	
		CC Homozygotes (*n* = 23) Mean ± SD	T-Allele Carriers (*n* = 20) Mean ± SD	2.5 g/d (*n* = 24) Mean ± SD	5.0 g/d (*n* = 19) Mean ± SD
TAG (mg/dL)	BET_pre_	93.6 ± 36.5	105.5 ± 36.8	99.4 ± 44.1	98.5 ± 38.6
	BET_post_	92.9 ± 39.9	121.8 ± 57.2	104.5 ± 57.8	107.9 ± 38.9
	PL_pre_	104.9 ± 53.0	119.7 ± 54.1	114.1 ± 58.2	108.3 ± 47.7
	PL_post_	100.7 ± 48.6	108.1 ± 58.1	106.8 ± 54.0	100.3 ± 51.9
*Time x* *treatment*	*p* = 0.069 η^2^ = 0.084	*Time x treatment x MTHFR:*	*p* = 0.153 η^2^ = 0.053	*Time x treatment x dose:*	*p* = 0.729 η^2^ = 0.003
TC (mg/dL)	BET_pre_	191.7 ± 31.9	192.5 ± 32.2	194.3 ± 33.1	189.1 ± 30.3
	BET_post_	193.4 ± 47.1	192.7 ± 30.2	194.9 ± 43.8	190.7 ± 35.2
	PL_pre_	189.8 ± 28.0	195.4 ± 27.9	197.5 ± 31.5	185.5 ± 20.7
	PL_post_	184.6 ± 25.8	191.3 ± 27.1	191.7 ± 29.5	182.2 ± 21.0
*Time x* *treatment*	*p* = 0.247 η^2^ = 0.035	*Time x treatment x MTHFR:*	*p* = 0.764 η^2^ = 0.002	*Time x treatment x dose:*	*p* = 0.859 η^2^ = 0.001
LDL-C (mg/dL)	BET_pre_	113.3 ± 23.6	113.3 ± 27.6	116.4 ± 24.4	109.2 ± 26.4
	BET_post_	115.4 ± 33.3	112.7 ± 26.3	117.5 ± 30.1	109.7 ± 30.2
	PL_pre_	111.6 ± 18.3	115.5 ± 24.3	118.2 ± 22.4	106.9 ± 17.7
	PL_post_	109.2 ± 19.5	111.0 ± 22.5	113.7 ± 20.9	105.1 ± 19.9
*Time x* *treatment*	*p* = 0.295 η^2^ = 0.029	*Time x treatment x MTHFR:*	*p* = 0.915 η^2^ = 0.000	*Time x treatment x dose*:	*p* = 0.642 η^2^ = 0.006
HDL-C (mg/dL)	BET_pre_	58.1 ± 14.2	56.1 ± 13.1	56.1 ± 13.3	58.7 ± 14.1
	BET_post_	57.6 ± 15.9	55.3 ± 11.0	55.5 ± 15.5	58.0 ± 11.5
	PL_pre_	55.8 ± 12.1	54.4 ± 12.4	55.6 ± 12.6	54.6 ± 11.7
	PL_post_	56.7 ± 13.7	58.0 ± 13.9	57.0 ± 13.0	57.7 ± 14.6
*Time x* *treatment*	*p* = 0.077 η^2^ = 0.080	*Time x treatment x MTHFR:*	*p* = 0.325 η^2^ = 0.025	*Time x treatment x dose:*	*p* = 0.552 η^2^ = 0.009
Glucose (mg/dL)	BET_pre_	87.2 ± 9.5	89.2 ± 6.7	87.5 ± 6.6	88.8 ± 10.3
	BET_post_	89.3 ± 8.2	89.2 ± 9.0	89.3 ± 9.3	89.2 ± 7.4
	PL_pre_	89.1 ± 8.5	87.7 ± 8.1	88.0 ± 8.8	89.1 ± 7.7
	PL_post_	88.8 ± 9.7	90.0 ± 6.0	89.0 ± 6.9	89.4 ± 9.8
*Time x* *treatment*	*p* = 0.928 η^2^ = 0.000	*Time x treatment x MTHFR:*	*p* = 0.276 η^2^ = 0.031	*Time x treatment x dose:*	*p* = 0.816 η^2^ = 0.001
Homocysteine (μmol/L)	BET_pre_	16.6 ± 3.5	17.6 ± 4.6	17.3 ± 4.2	16.8 ± 3.9
	BET_post_	15.0 ± 3.1	16.2 ± 3.9	15.6 ± 3.8	15.6 ± 3.3
	PL_pre_	15.5 ± 2.4	17.8 ± 5.1	16.6 ± 4.4	16.7 ± 3.7
	PL_post_	15.5 ± 3.1	17.4 ± 4.9	17.2 ± 4.1	17.7 ± 6.0
*Time x* *treatment*	*p* = 0.009 η^2^ = 0.164	*Time x treatment x MTHFR:*	*p* = 0.446 η^2^ = 0.015	*Time x treatment x dose:*	*p* = 0.888 η^2^ = 0.001

BET_post_, after betaine supplementation; BET_pre_, before betaine supplementation; HDL-C, high-density lipoprotein cholesterol; LDL-C, low-density lipoprotein cholesterol; MTHFR, methylene tetrahydrofolate reductase; PL_post_, after placebo supplementation; PL_pre_, before placebo supplementation; TAG, triacylglycerol; TC, total cholesterol.

**Table 5:** The effect of betaine supplementation on the concentrations of cytokines in relation to *MTHFR* genotype and BET dose.

		*MTHFR*		Dose	
		CC Homozygotes (*n* = 23) Mean ± SD	T Allele-Carriers (*n* = 20) Mean ± SD	2.5 g/d (*n* = 24) Mean ± SD	5.0 g/d (*n* = 19) Mean ± SD
IL-6 (pg/mL)	BET_pre_	2.22 ± 0.88	2.29 ± 0.23	2.31 ± 0.85	2.17 ± 0.25
	BET_post_	2.10 ± 0.35	2.17 ± 0.29	2.11 ± 0.31	2.17 ± 0.35
	PL_pre_	2.20 ± 0.45	2.23 ± 0.21	2.13 ± 0.29	2.31 ± 0.41
	PL_post_	2.09 ± 0.26	2.36 ± 0.49	2.20 ± 0.31	2.24 ± 0.50
*Time x* *treatment*	*p* = 0.400 η^2^ = 0.018	*Time x treatment x MTHFR:*	*p* = 0.290 η^2^ = 0.029	*Time x treatment x dose:*	*p* = 0.184 η^2^ = 0.045
IL-1β (pg/mL)	BET_pre_	2.51 ± 0.39	2.50 ± 0.16	2.53 ± 0.35	2.47 ± 0.23
	BET_post_	2.44 ± 0.25	2.56 ± 0.21	2.53 ± 0.21	2.45 ± 0.25
	PL_pre_	2.46 ± 0.22	2.45 ± 0.15	2.47 ± 0.15	2.43 ± 0.23
	PL_post_	2.53 ± 0.30	2.51 ± 0.22	2.53 ± 0.29	2.51 ± 0.22
*Time x* *treatment*	*p* = 0.221 η^2^ = 0.038	*Time x treatment x MTHFR:*	*p* = 0.262 η^2^ = 0.032	*Time x treatment x dose:*	*p* = 0.768 η^2^ = 0.002
TNF-α (pg/mL)	BET_pre_	3.64 ± 0.93	3.33 ± 0.87	3.44 ± 0.87	3.56 ± 0.96
	BET_post_	3.73 ± 0.82	3.36 ± 0.87	3.48 ± 0.85	3.65 ± 0.87
	PL_pre_	3.42 ± 0.82	3.31 ± 0.89	3.43 ± 0.85	3.28 ± 0.85
	PL_post_	3.42 ± 0.78	3.29 ± 0.92	3.45 ± 0.89	3.25 ± 0.79
*Time x* *treatment*	*p* = 0.407 η^2^ = 0.019	*Time x treatment x MTHFR:*	*p* = 0.907 η^2^ = 0.000	*Time x treatment x dose:*	*p* = 0.530 η^2^ = 0.011

BET_post_, after betaine supplementation; BET_pre_, before betaine supplementation; IL, interleukin; *MTHFR*, methylene tetrahydrofolate reductase; PL_post_, after placebo supplementation; PL_pre_, before placebo supplementation; TNF-α, tumour necrosis factor α.

**Table 6:** The effect of betaine supplementation on liver enzymes and its relation to *MTHFR* genotype and BET dose.

		*MTHFR*		Dose	
		CC Homozygotes (*n* = 23) Mean ± SD	T-Allele Carriers (*n* = 20) Mean ± SD	2.5 g/d (*n* = 24) Mean ± SD	5.0 g/d (*n* = 19) Mean ± SD
ALAT (U/L)	BET_pre_	36.2 ± 36.4	30.5 ± 11.1	34.5 ± 34.4	32.3 ± 16.2
	BET_post_	30.2 ± 12.6	31.5 ± 12.8	29.6 ± 10.8	32.3 ± 14.7
	PL_pre_	34.7 ± 23.1	32.0 ± 13.7	35.2 ± 20.6	31.4 ± 17.5
	PL_post_	33.2 ± 20.8	33.8 ± 15.2	33.4 ± 17.1	33.6 ± 20.0
*Time x* *treatment*	*p* = 0.617 η^2^ = 0.007	*Time x treatment x MTHFR:*	*p* = 0.746 η^2^ = 0.003	*Time x treatment x dose:*	*p* = 0.959 η^2^ = 0.000
ASPAT (U/L)	BET_pre_	34.9 ± 24.6	31.6 ± 8.7	31.9 ± 19.6	35.2 ± 18.2
	BET_post_	29.1 ± 7.9	32.1 ± 11.5	28.9 ± 5.5	32.6 ± 13.2
	PL_pre_	33.7 ± 16.6	32.5 ± 9.1	33.0 ± 11.6	33.2 ± 15.9
	PL_post_	34.1 ± 15.5	36.9 ± 20.8	36.5 ± 21.0	34.0 ± 13.7
*Time x* *treatment*	*p* = 0.193 η^2^ = 0.043	*Time x treatment x MTHFR:*	*p* = 0.768 η^2^ = 0.002	*Time x treatment x dose:*	*p* = 0.661 η^2^ = 0.005

ALAT, alanine transferase; ASPAT, aspartate transferase; BET_post_, after betaine supplementation; BET_pre_, before betaine supplementation; *MTHFR*, methylene tetrahydrofolate reductase; PL_post_, after placebo supplementation; PL_pre_, before placebo supplementation.

## Data Availability

The data supporting reported results are available on request from the corresponding author (A.C.).
